# Increased Risk of American Tegumentary Leishmaniasis in an Urban and Rural Area of Caratinga, Brazil between 2016 and 2021

**DOI:** 10.4269/ajtmh.23-0017

**Published:** 2023-08-28

**Authors:** Rafael L. Neves, Fabrício T. O. Ker, Felipe Dutra-Rêgo, Jeronimo M. N. Rugani, José D. Andrade Filho, Rodrigo P. Soares, Célia M. F. Gontijo

**Affiliations:** ^1^ René Rachou Institute, Oswaldo Cruz Foundation, Belo Horizonte, Brazil;; ^2^ Laboratory of Epidemiology of Infectious and Parasitic Diseases, Institute of Biological Sciences, Federal University of Minas Gerais, Belo Horizonte, Brazil

## Abstract

We used spatial analysis tools to examine the epidemiological situation and spatial distribution of American tegumentary leishmaniasis in the municipality of Caratinga between 2016 and 2021. In addition, potential sandfly vectors were captured. All information used in this study was retrieved from public health archives and confirmed in the state health services databases. All cases were analyzed using Geographic Information Systems software. In addition, sandfly collections and molecular detection of *Leishmania* were carried out in areas with the highest number of cases. During the analyzed period, American tegumentary leishmaniasis (ATL) cases increased and remained high in the last years. The hotspots included urban areas of Caratinga city and the districts of Patrocínio of Caratinga and Sapucaia. The species *Nyssomyia whitmani*, *Nyssomyia intermedia*, and *Migonemyia migonei* were the most abundant species and the ITS1-polymerase chain reaction technique detected *Leishmania* DNA in these species. On the basis of our analyses, the urbanization of ATL in Caratinga has taken place in recent years. Because of the increase in the number of human cases and the presence of vectors, it is recommended that health authorities focus on control measures in hotspots.

## INTRODUCTION

American tegumentary leishmaniasis (ATL) is a zoonoses related to land use and biodiversity management and often related to occupational exposure.^1^ In recent decades, visceral leishmaniasis urbanization has been increasingly reported in several cities, especially in Brazil as a result of the adaptation of the main vector *Lutzomyia longipalpis* to the urban areas.^2^^,^^3^ However, the epidemiological aspects of ATL transmission are much more complex depending on the *Leishmania* species, vectors adaptability, and ecological situations. Although most studies have shown a correlation between gender and occupational exposure in several countries, this characteristic may not be clear in others.^2^

The municipality of Caratinga, located in Rio Doce Valley, east of the state of Minas Gerais, Brazil, urban characteristics surrounded by rural areas of coffee crops, where a high number of ATL cases are reported in occupationally exposed workers.^4^^,^^5^ The main sandflies captured in these areas are *Nyssomyia whitmani* and *Migonemyia migonei*.^6^ Studies published in the 1970s^6–8^ and in 2021^5^ showed that Caratinga is an ATL transmission area with rural and recent urban cases of the disease by *Leishmania braziliensis*. In our previous study,^5^ those cases were in the north and southwest regions of the city overlapping with ATL proven vectors. This finding is of epidemiological relevance because of the urbanization of ATL is less common than visceral leishmaniasis.

Geographic Information Systems approaches and spatial analysis methods are useful tools in several Latin American countries and Amazonian regions.^2^^,^^3^^,^^9^ As a part of a wider study on ATL in Caratinga, the aim of our work was to confirm its urban transmission by reporting an additional 3-year period (2019–2021) follow-up. In our previous work, we provided preliminary data on sandfly captures.^8^ Here, a detailed ecological analysis using spatial and molecular approaches is provided to ascertain sandflies presence and infection in rural and urban sites. Our main hypothesis was to ascertain urbanization establishment in Caratinga in recent years (2016–2021) and to detect *Leishmania* in the sandflies in all areas surveyed.

## MATERIALS AND METHODS

### Strategy for data analysis.

Data were analyzed using methodology reported previously,^5^ with modifications. It is important to note that we added another 3-year period (2019–2021) to the previous historical series (2007–2018). In this article, we compared the last triennium (2016–2018) of the historical series with 2019–2021 period. However, in some analyses, we referred to data between 2007 and 2015 for better picture along time. Bayesian empirical spatial analysis^10^ estimated incidence rates for each 3-year period using GeoDa software version 1.14 (ASU, GeoDa Center for Geospatial Analysis and Computation, Tempe, AZ). Standard Deviational Ellipses^11^^,^^12^ and Kernel density spatial analysis were used to determine ATL distribution using ArcMap 10.6.1^13^ for the entire historical series and (2007–2021) the last two trienniums (2016–2018/2019–2021), respectively. Density maps of the trienniums were standardized in categories. Higher than zero (> 0) were successively overlapped to assess the development of the affected area and the number of positive trienniums. Finally, the relative risk (RR) technique was determined using SaTScan 9.4.4 software.^14^

### Study area.

**Figure 1.**
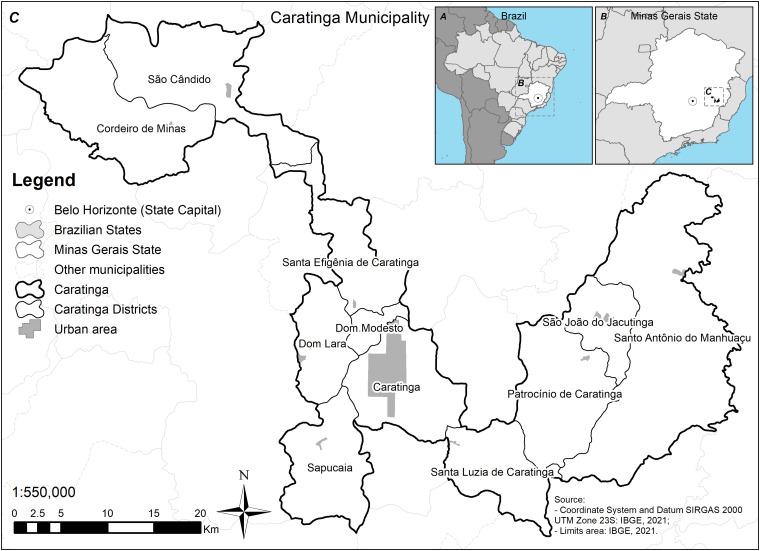
Geographic localization of municipality of Caratinga, Minas Gerais state, Brazil. (**A**) Localization of Brazil in South America. (**B**) Localization of Minas Gerais state in Brazil. (**C**) Municipality of Caratinga.

The study was carried out in the municipality of Caratinga (19°47′25″S and 42°8′21″W), Minas Gerais state, Brazil. Demographic information is reported elsewhere.^15^ Caratinga has 10 districts: Cordeiro de Minas, Dom Lara, Dom Modesto, Patrocínio de Caratinga, Santa Efigênia de Caratinga, Santa Luzia de Caratinga, Santo Antônio do Manhuaçu, Sapucaia, São Cândido, and São João Jacutinga (Figure 1).^16^

### Sandfly captures.

**Figure 2.**
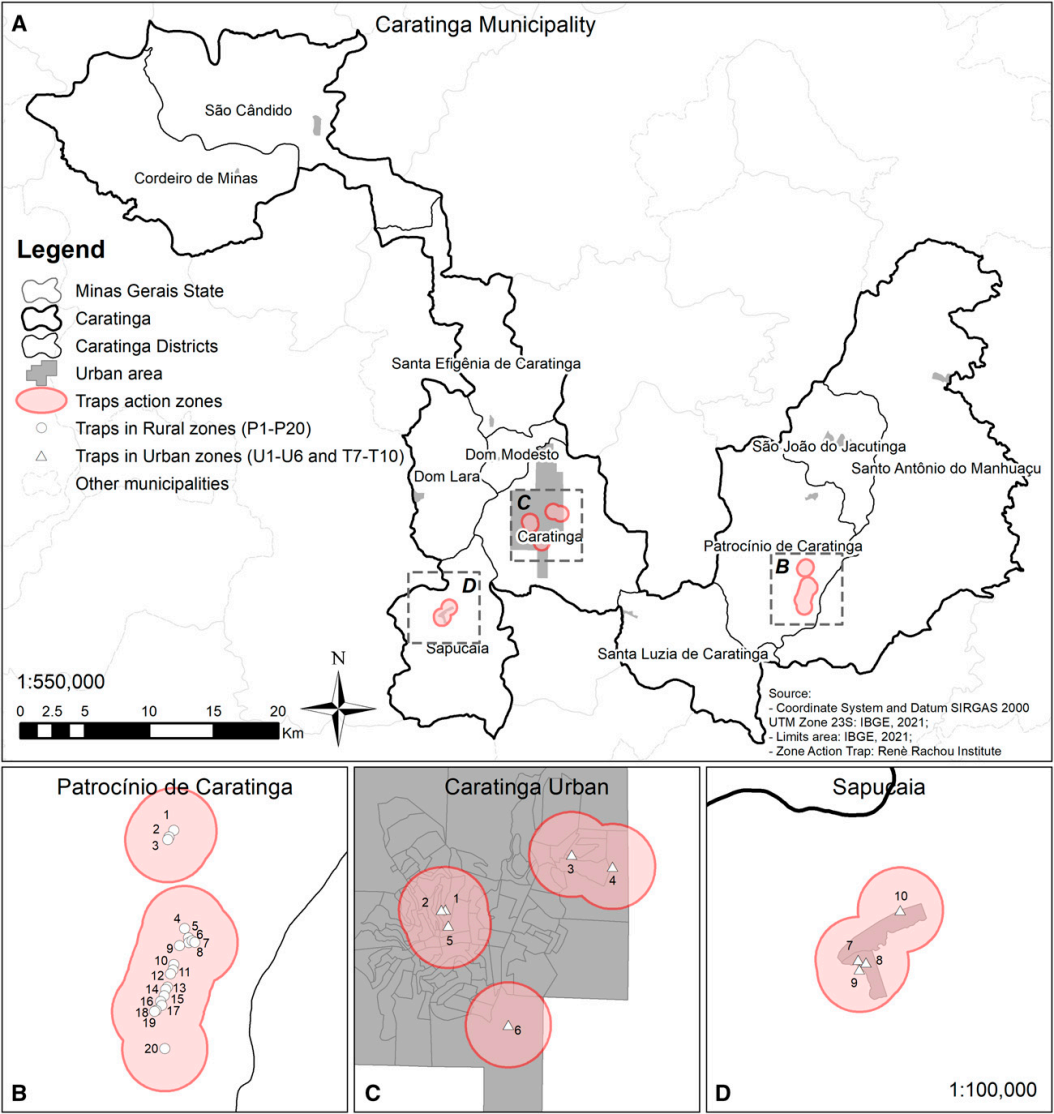
Collection points of sand fly in municipality of Caratinga (**A**), Patrocínio de Caratinga district (**B**), urban area of Caratinga (**C**), and Sapucaia district (**D**).

Entomological captures (2020–2021) used HP light traps^17^ were performed under a license for zoological material collection (SISBio #15237-2) (Figure 2A). Systematic captures occurred in January, March, May, July, September, and November. Those areas were chosen based on the highest number of ATL cases. In 2020, 20 collection points (P1–P20) were in Córrego Volta Grande (Patrocínio de Caratinga district) (Figure 2B). In 2021, 10 collection points were selected, six (U1–U6) in the urban area of Caratinga (Figure 2C), and four in the district of Sapucaia (T7–T10) (Figure 2D). Insects were stored in glycerin before identification and molecular approaches.

### Diversity, similarity, and hotspot assessment.

To evaluate sandfly fauna, species diversity indexes were calculated for each point and district including taxa richness (S), individual abundance (N), dominance (D), Simpson (1-D), Shannon–Weiner (H), equitability (J), and Margalef. H and 1-D indexes were compared between pairs of districts with *t* test. Similarity between districts and sampling points was evaluated using Jaccard^18^ and hierarchical clustering, respectively. Kernel analysis calculated the magnitude and adjusted distribution and abundance of ATL cases and infected vectors.

### Vectors and factors associated with human cases.

The probability associated with the occurrence of ATL cases (2007–2021) was estimated in relation to vector collections between 2020 and 2021. Although data on sandfly collection before 2020 were not available, this analysis helps to identify the hotspots by overlapping ATL cases and vectors during the entire period. The outcome or dependent variable was dichotomized according to the presence of ATL in 600-m radius of each point (*N* = 30) of vector collection: coded as 1 if the point had reported at least 1 case, or as 0 if none was confirmed.

### *Leishmania* detection and typing.

*Leishmania* detection was performed in captured sandflies and human patients. The latter was made from biopsies collected at the city health service (2020–2021). Polymerase chain reaction (PCR) targeted ITS1 gene before digestion with *Hae*III.^19^ Phlebotomine were tested with a minimum of one sandfly female specimen or pooled to a maximum of 10 female specimens of the same species, date and place of collection. Negative control groups used male sandflies. Positive controls with 20 nanograms of DNA extracted from reference strains of *L. amazonensis* (IFLA/BR/67/PH8), *L. braziliensis* (MHOM/BR/75/M2903), *L. infantum* (MHOM/BR/74/PP75), *L. guyanensis* (MHOM/BR/75/M4147), and *L. major* (MHOM/IL/81/Friedlin), were used. Positive samples were submitted to digestion by *Hae*III to identify *Leishmania* species.^20^ Amplified PCR products were sequenced. Reactions were performed using BigDye Terminator v.3.1 Cycle Sequencing (Applied Biosystems, Foster City, CA) following the manufacturer’s specifications and assayed in the Sanger ABI 3730 Sequencing. Consensus sequences were obtained and edited using the software package Phred/Phrap/Consed version: 0.020425.c (University of Washington, Seattle, WA). The sequences were evaluated against NCBI database using BLASTn.^21^

### Identification of blood meal sources in blood-fed sandfly females.

The identification of blood gut content of engorged sandfly females was conducted by PCR-based analysis of the cytochrome B gene, using primers previously described,^22^ followed by sequencing the 383-bp product. The procedure was performed following the protocol described by Carvalho et al.^22^ Amplicons were removed from the agarose gel purified by a QIAquick Gel Extraction Kit (Qiagen, Chatsworth, CA) and sequenced with ABI PRISM 3730XL DNA Analyzer (Applied Biosystems). FinchTV software (Geospiza Inc., Seattle, WA) was used for manual verification of the electropherograms and SeqTrace software^23^ was used to align and calculate consensus sequences for regions of similarity with GenBank sequences by the Basic Local Alignment Search Tool.

## RESULTS

The incidence rate of ATL cases in the period 2007–2021 varied. The lowest rate was observed in 2007 (0.72) and the highest in 2010 (6.57) (Table 1). In the past two trienniums (2016–2018 and 2019–2021), the incidence in the municipality of Caratinga was high but similar (13.82 versus 11.77), respectively. Interestingly, from 2007 to 2018 (12 years), 42 (13.17%) cases were registered in the urban area, whereas there were 31 (28.45%) notifications in only the past 3 years (2019–2021).

**Table 1:** Number of cases and incidence per 10,000 inhabitants of ATL and population of the municipality of Caratinga from 2007 to 2021

Year	*N*	Population	Incidence	Incidence per triennium
2007	6	83.363	0.72	5.06
2008	19	84.825	2.24	
2009	18	85.469	2.11	
2010	56	85.239	6.57	11.88
2011	39	85.811	4.54	
2012	7	86.364	0.81	
2013	12	89.578	1.34	5.21
2014	9	90.192	1.00	
2015	26	90.782	2.86	
2016	41	91.342	4.49	13.82
2017	39	91.841	4.25	
2018	47	91.503	5.14	
2019	44	92.062	4.78	11.77
2020	46	92.603	4.97	
2021	19	93.124	2.04	
Total	428		4.74	

ATL = American tegumentary leishmaniasis.

Although we did not see a substantial difference between the total number of ATL cases in the last two trienniuns (Table 1), we detected variations in some districts (Table 2). For example, in Patrocício de Caratinga, although there was a decrease in the number of cases from 2016 to 2021, this district still contributes with most of the incidence of cases (33.05%). Most of the districts and the urban area of Caratinga had an increase in the ATL cases during this period including: Cordeiro de Minas, Dom Modesto, Santa Efigênia de Caratinga, Santa Luzia de Caratinga, Santo Antônio de Manhuaçu, and São Cândido (Table 2).

**Table 2:** Number of ATL cases by city/district during 2016–2021

District	2016–2018	2019–2021	2016–2021	%
Caratinga	20	25	45	19.07
Cordeiro de Minas	2	3	5	2.12
Dom Lara	2	2	4	1.69
Dom Modesto	4	7	11	4.66
Patrocínio de Caratinga	57	21	78	33.05
Santa Efigênia de Caratinga	4	7	11	4.66
Santa Luzia de Caratinga	9	15	24	10.17
Santo Antônio do Manhuaçu	5	10	15	6.36
São Cândido	1	2	3	1.27
São João do Jacutinga	7	4	11	4.66
Sapucaia	16	13	29	12.29
Total	127	109	236	100

ATL = American tegumentary leishmaniasis.

**Figure 3.**
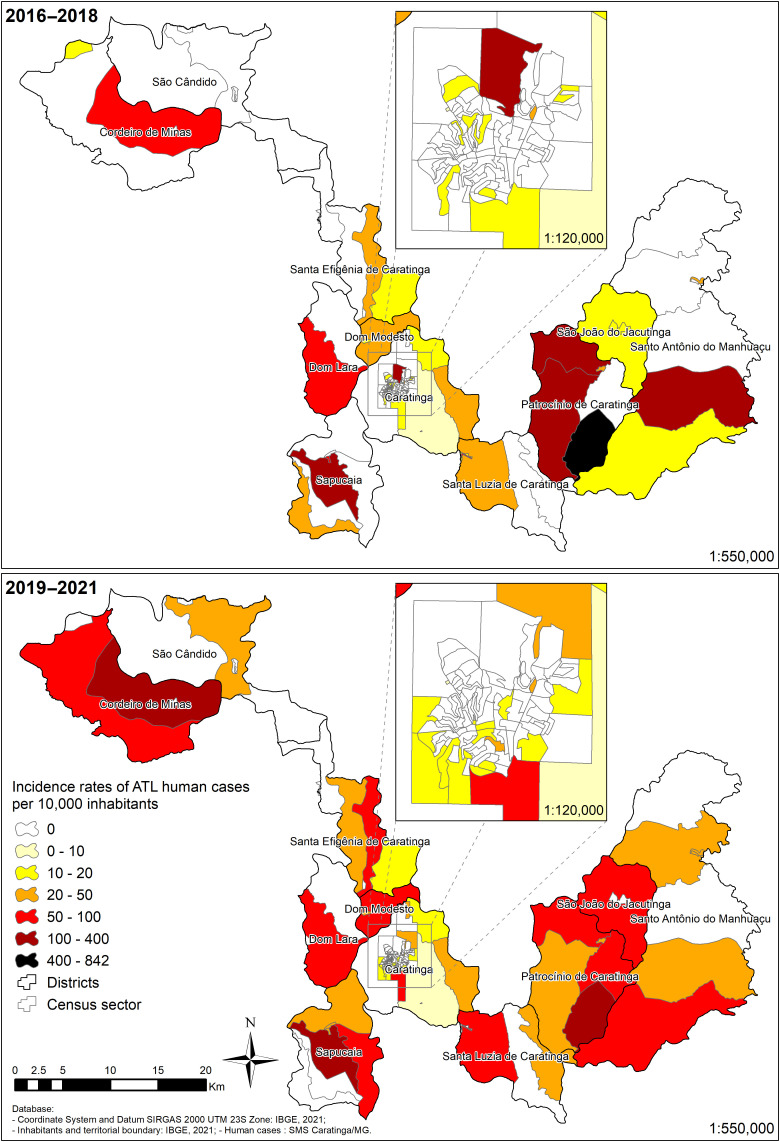
Incidence rates of American tegumentary leishmaniasis urban and rural human cases accumulated per 10,000 inhabitants in the municipality of Caratinga between 2016–2018 and 2019–2021.

**Figure 4.**
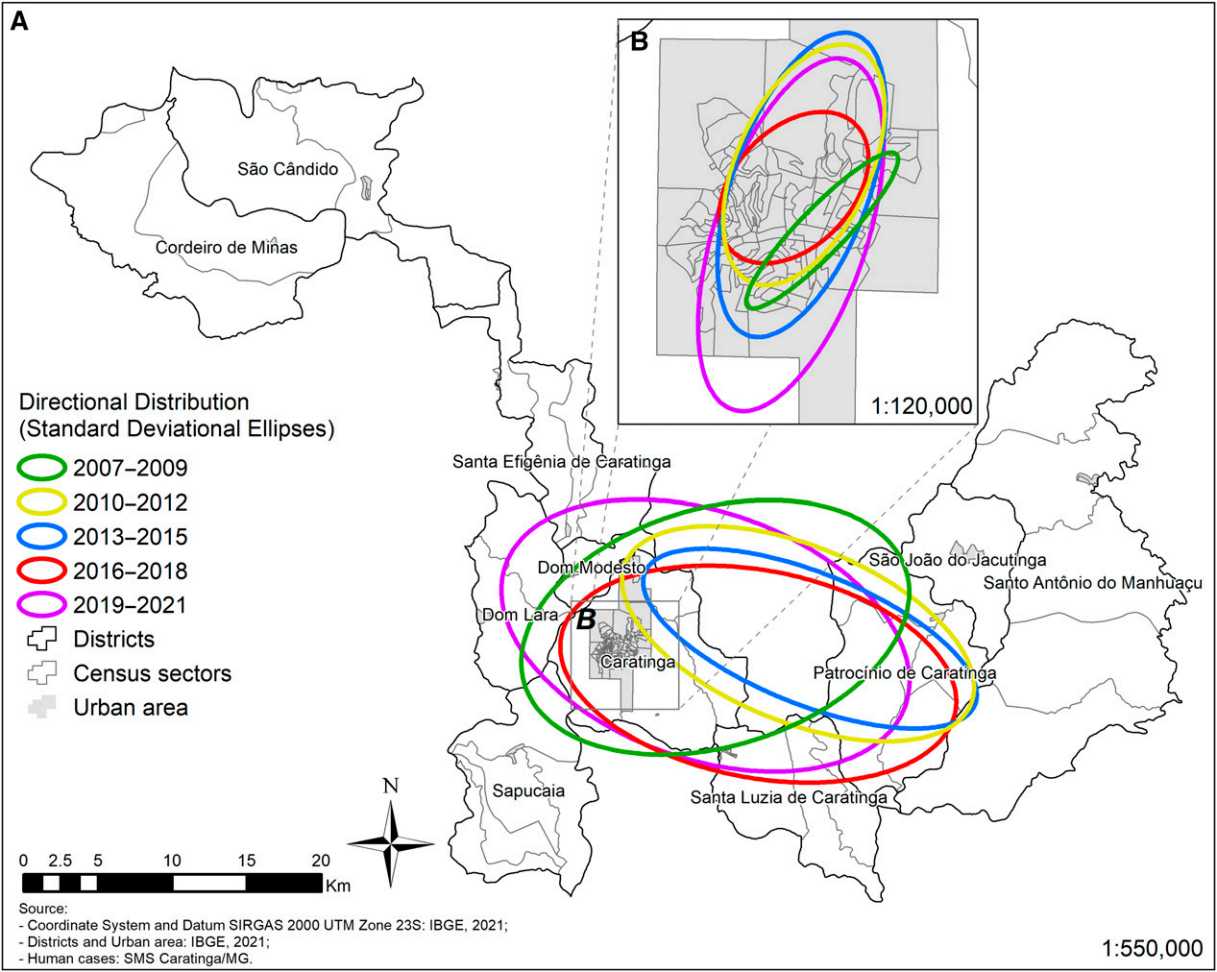
Directional distribution ellipses of American tegumentary leishmaniasis cases in municipality of Caratinga (**A**) and urban area of Caratinga (**B**).

During the period 2019–2021, incidence rates of up to 255.1/10,000 inhabitants per census sector were detected, with urban and rural ATL cases in all districts. Notably, not only the distribution but also the incidence has increased to other urban and rural areas (Figure 3). Except for 2010–2012/2013–2015 trienniums, which had similar ellipses distribution, the 2019–2021 ellipse was wider expanding in all directions with a slight shift northward (Figure 4A). In the urban area (Figure 4B) during 2010–2015, there was a slight increase in the amplitude of the ellipses followed by a decrease in 2016–2018. The 2019–2021 ellipse spanning part of the north/south axis.

**Figure 5.**
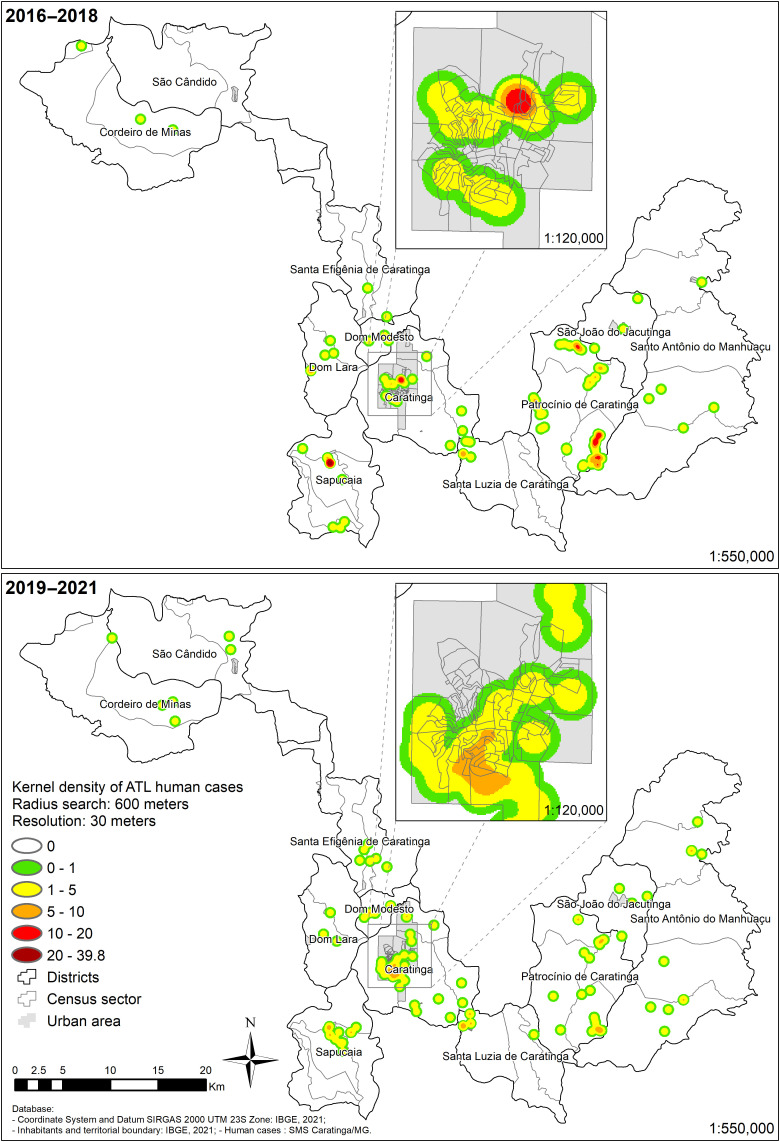
Kernel density of American tegumentary leishmaniasis urban and rural human cases in the municipality of Caratinga between 2016–2018 and 2019–2021.

Kernel density maps (2019–2021) showed that in 2016–2018, there was a higher concentration of cases (*n* = 40). In 2019–2021, there was a reduction (up to 10 cases) (Figure 5). However, they comprised a higher number of hotspots and occupied a larger area (82 km^2^, historical record) (Table 3).

**Table 3:** Affected area by triennium in municipality of Caratinga

Triennium	Affected area (km^2^)	Increase (km^2^)	Accumulated affected area (km^2^)
2007–2009	39	–	39*
2010–2012	66	+56	95*
2013–2015	34	+15	110*
2016–2018	68	+29	139
2019–2021	82	+32	171

* Data from Neves et al.^5^

**Figure 6.**
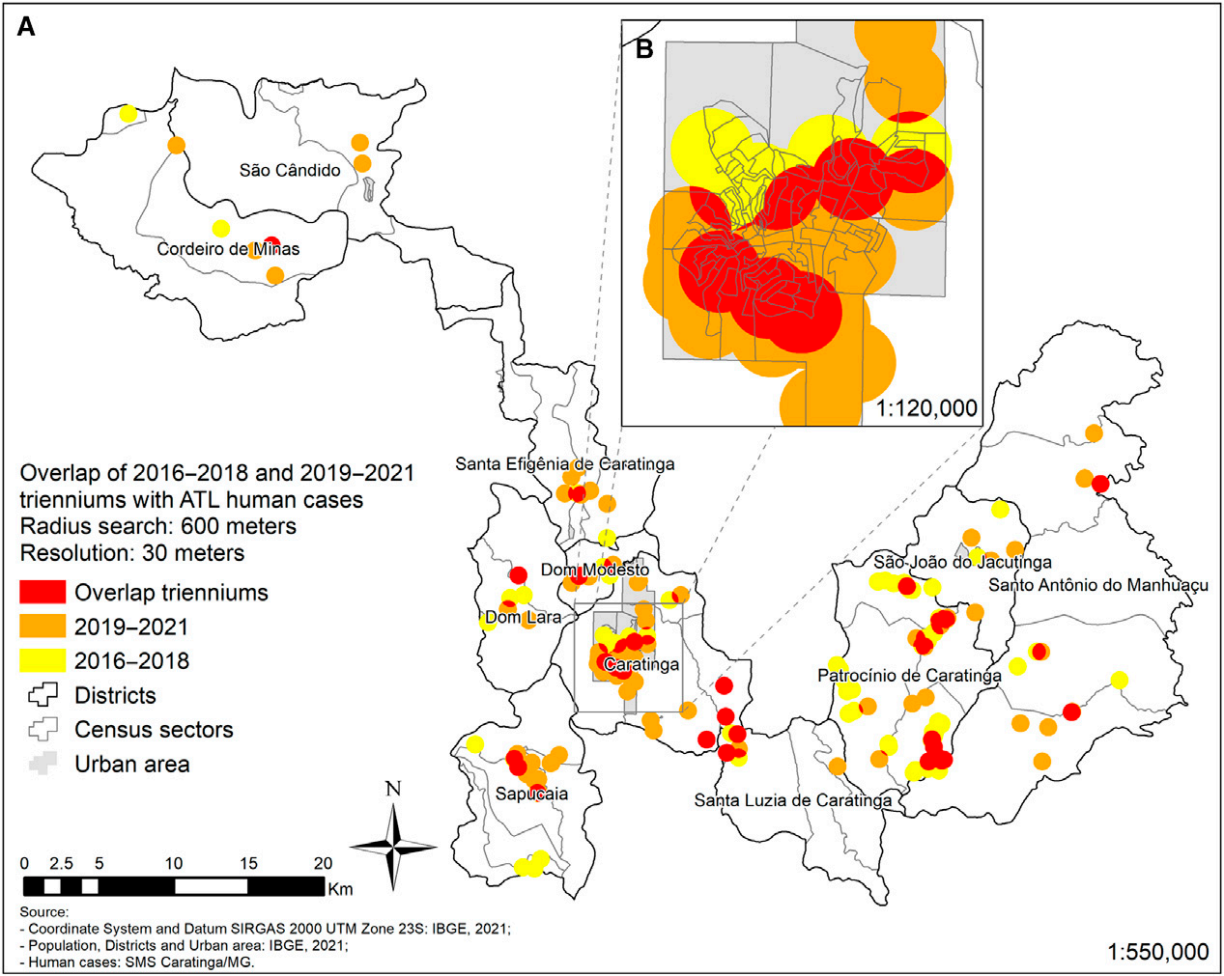
American tegumentary leishmaniasis human cases in the municipality of Caratinga (**A**) and urban area of Caratinga (**B**) between 2016–2018 and 2019–2021.

Consistent with the ellipses analysis, a total coverage area of 171 km^2^ showed an expansion of the disease (Table 3). This pattern was also seen in the urban area of Caratinga, where the number of cases expanded southwest (Figure 5), and in the districts of Patrocínio de Caratinga, Sapucaia, Santa Efigênia (Figure 6). The situation is more critical in the urban area of Caratinga, where areas of high frequency of ATL occur in the northeast, center, and south regions.

**Figure 7.**
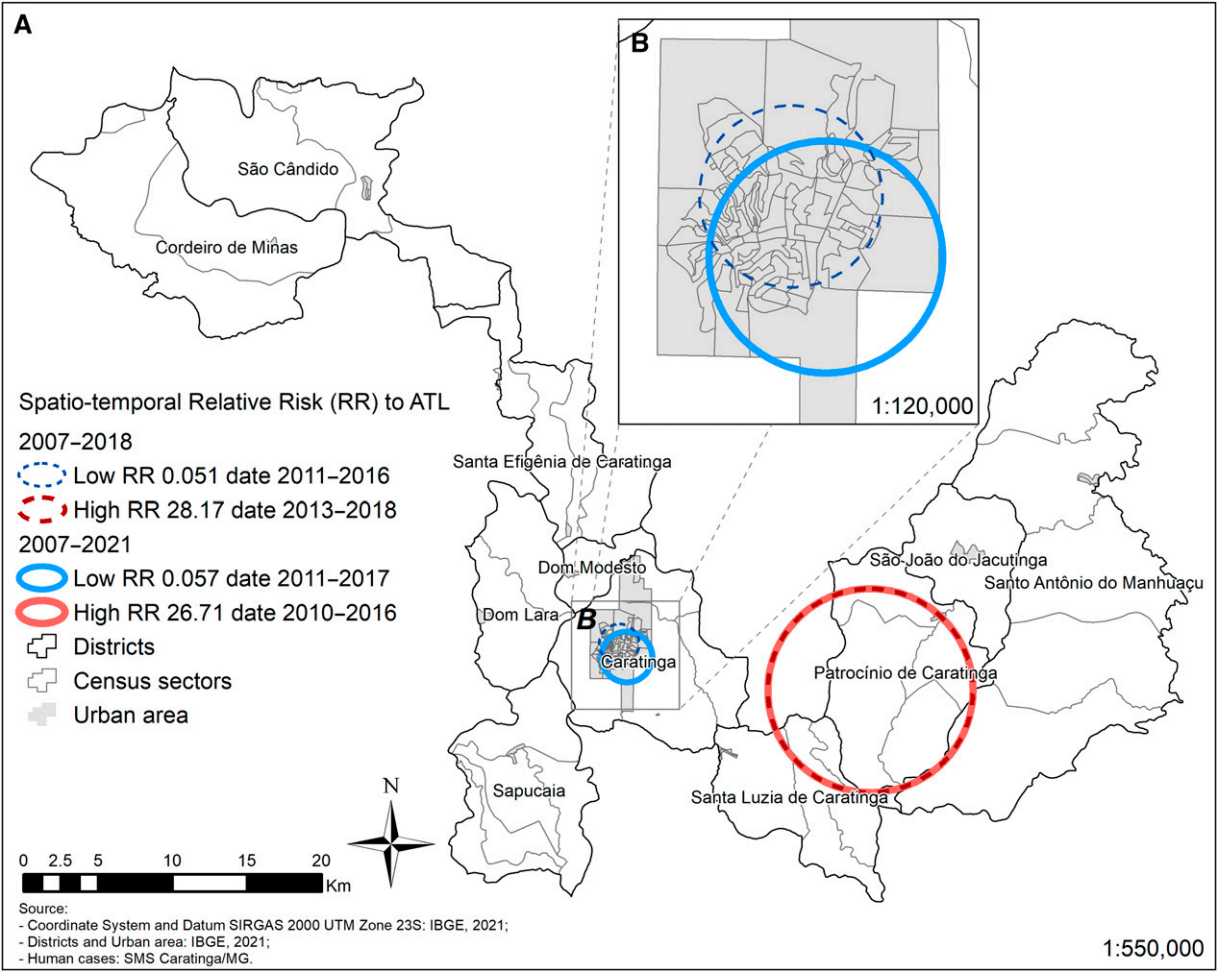
Spatiotemporal relative risk to American tegumentary leishmaniasis during the period studies in the municipality of Caratinga (**A**) and urban area of Caratinga (**B**).

The analysis of RR was carried out in two periods (2007–2018 and 2007–2021) (Figure 7). The RRs were similar in both periods analyzed. Highest risks (28.17 and 26.71) were detected in the rural area of Patrocínio de Caratinga, respectively. Lowest risks (0.051 and 0.057) were found in the urban area of Caratinga.

**Figure 8.**
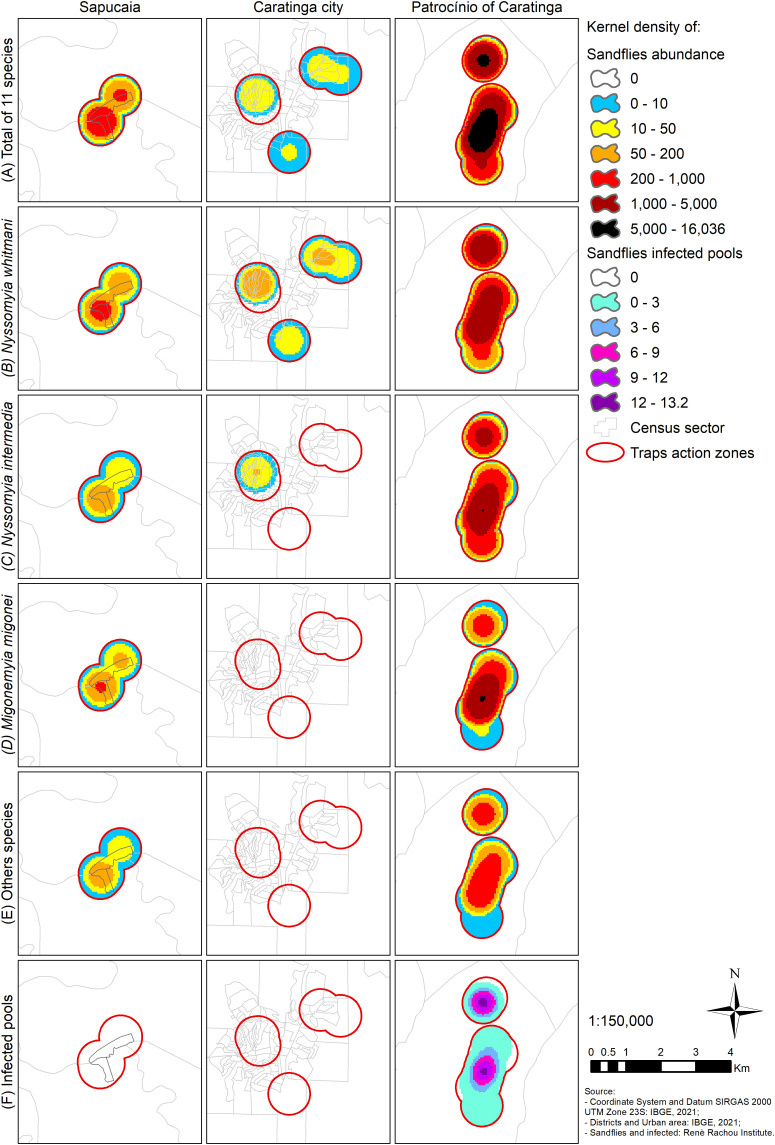
Total sandflies collected (**A**), total *Nyssomyia whitmani* species (**B**), total *Nyssomyia intermedia* species (**C**), total *Migonemyia migonei* species (**D**), total others species (**E**), and total sandflies infected pools (**F**).

A total of 13,043 sandfly specimens from seven genera, and 11 species were collected, 7,547 females (57.86%) and 5,496 males (42.14%) including 5,235 (40.14%) *Nyssomyia whitmani*, 3,948 (30.27%) *Nyssomyia intermedia*, 3,078 (23.60%) *Migonemyia migonei*, 238 (1.82%) *Evandromyia lenti*, 218 (1.67%) *Pintomyia pessoai*, 153 (1.17%) *Micropygomyia quinquefer,* 108 (0.83%) *Nyssomyia neivai*, 57 (0.44%) *Evandromyia cortelezzii*, 3 (0.02%) *Brumptomyia cunhai*, 3 (0.02%) *Sciopemyia microps*, and 2 (∼0.02%) *Micropygomyia capixaba* (Table 4). The total density of sandflies collected is provided in Figure 8A). *Ny. whitmani* (Figure 8B), *Ny. intermedia* (Figure 8C), and *Mg. migonei* (Figure 8D) were the most abundant (Figure 8E). The density of sandflies was higher in Patrocínio de Caratinga and lower in Caratinga city.

**Table 4:** Number of sandflies collected per year of collection in the municipality of Caratinga

Species	2020 Patrocínio de Caratinga			2021 Caratinga (urban area) and Sapucaia			Total			
	♂	♀	Total	♂	♀	Total	♂	♀	Total	Total (%)
*Brumptomyia cunhai*	3	0	3	0	0	0	3	0	3	0.02
*Evandromyia cortelezzii*	0	0	0	20	37	57	20	37	57	0.44
*Evandromyia lenti*	100	138	238	0	0	0	100	138	238	1.82
*Micropygomyia quinquefer*	11	142	153	0	0	0	11	142	153	1.17
*Micropygomyia capixaba*	2	0	2	0	0	0	2	0	2	0.02
*Migonemyia migonei*	1,372	1,591	2,963	51	64	115	1,423	1,655	3,078	23.60
*Nyssomyia intermedia*	1,515	2,335	3,850	44	54	98	1,559	2,389	3,948	30.27
*Nyssomyia neivai*	40	68	108	0	0	0	40	68	108	0.83
*Nyssomyia whitmani*	2,097	2,827	4,924	143	168	311	2,240	2,995	5,235	40.14
*Pintomyia pessoai*	98	120	218	0	0	0	98	120	218	1.67
*Sciopemyia microps*	0	3	3	0	0	0	0	3	3	0.02
Total (%)	5,238	7,224	12,462	258	323	581	5,496	7,547	13,043	100
	42.03	57.97	100	44.41	55.59	100	42.14	57.86		

Low species diversity was found in the urban area of Caratinga, represented by vectors as shown by the high dominance (D) index, whereas the Simpson (1-D) and Shannon (H) indexes were very low. In each Caratinga point, the richness varied between 2 and 10 species, and the highest Shannon and Simpson indexes were 1.318 and 0.702, respectively (Table 6). In the urban area of Caratinga, the H and 1-D diversity indexes were lower than in the other locations. As observed, the districts of Sapucaia and Patrocínio de Caratinga presented higher richness (S) and abundance (N), and dominance (D) was low. Diversity indexes were similar between areas and higher than in the city of Caratinga but still considered too low for the sampling effort, as corroborated by Margalef’s index. The equitability (J) was high in Sapucaia, and median in the city of Caratinga/Patrocínio de Caratinga, indicating a slight predominance of some species (Table 5).

**Table 5:** Diversity indices of vector species in the municipality of Caratinga

Index	Caratinga (urban area)	Sapucaia	Patrocínio de Caratinga	Total
Taxa (S)	2	4	10	11
Individuals (N)	149	432	12,462	13,043
Dominance (D)	0.768	0.298	0.309	0.309
Simpson (1-D)	0.232	0.702	0.691	0.691
Shannon (H)	0.394	1.293	1.318	1.331
Margalef	0.199	0.494	0.954	1.055
Equitability (J)	0.569	0.933	0.572	0.555

**Table 6:** Blood meal sources identified from sandfly females collected in the municipality of Caratinga, during 2020–2021

Species	Blood meal	Total score	Query cover (%)	E-value	Identity (%)
*Evandromyia lenti*	*Homo sapiens*	468	100	2 e-127	94.16
*Migonemyia migonei*	*Homo sapiens*	431	100	6 e-116	90.85
	*Homo sapiens*	573	100	8 e-159	99.69
	*Homo sapiens*	466	100	7 e-127	95.93
	*Homo sapiens*	383	100	8 e-102	87.94
	*Homo sapiens*	500	97	4 e-137	94.44
	*Homo sapiens*	567	98	3 e-157	99.38
	*Gallus gallus*	513	97	7 e-141	95.18
	*Gallus gallus*	512	100	2 e-140	96.55
	*Homo sapiens*	573	100	8 e-159	99.69
	*Gallus gallus*	532	100	2 e-146	99.66
*Nyssomyia intermedia*	*Homo sapiens*	555	100	3 e-153	99.36
	*Homo sapiens*	645	94	6 e-156	100.00
	*Homo sapiens*	570	100	1 e-157	99.69
	*Sus scrofa*	950	100	2 e-158	98.49
	*Sus scrofa*	896	100	4 e-154	99.06
	*Bos taurus*	572	97	3 e-158	98.79
	*Sus scrofa*	833	96	9 e-155	99.37
	*Homo sapiens*	444	100	3 e-120	91.46
	*Homo sapiens*	571	98	3 e-158	99.38
	*Sus scrofa*	778	100	8 e-156	99.37
	*Gallus gallus*	567	96	3 e-157	100.00
	*Sus scrofa*	535	100	2 e-147	98.38
	*Sus scrofa*	575	96	2 e-159	99.69
	*Sus scrofa*	349	100	1 e-91	90.64
*Nyssomyia whitmani*	*Sus scrofa*	851	100	3 e-155	99.68
	*Gallus gallus*	562	97	1 e-155	99.38
	*Gallus gallus*	428	96	2 e-115	89.57
	*Gallus gallus*	524	96	4 e-144	96.85
	*Gallus gallus*	535	95	2 e-147	97.77
	*Homo sapiens*	536	95	6 e-148	99.35
	*Homo sapiens*	542	98	1 e-149	98.72
	*Sus scrofa*	852	98	8 e-150	98.14
	*Sus scrofa*	872	100	2 e-137	95.56
	*Bos taurus*	527	95	3 e-145	96.88
	*Capra hircus*	687	98	8 e-160	99.69
	*Sus scrofa*	824	90	4 e-154	99.07
	*Gallus gallus*	478	100	1 e-129	96.31
	*Gallus gallus*	559	100	3 e-154	99.07
	*Homo sapiens*	581	97	2 e-161	100.00

**Figure 9.**
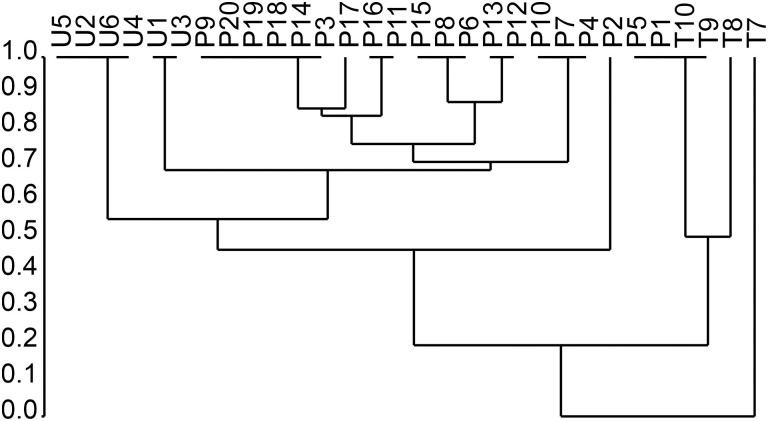
Similarity of sandflies collected in urban area of Caratinga (U1–U6), rural area of Patrocínio de Caratinga district (P1–P20), and transitional area of Sapucaia district (T7–T10).

The Jaccard Index showed low similarity between the evaluated areas, being 40% between the city of Caratinga and Sapucaia, 30% between Sapucaia and Patrocínio de Caratinga, and only 20% between the city of Caratinga and Patrocínio de Caratinga. The hierarchical grouping (Figure 9) showed the presence of three clusters: one each in the city of Caratinga (U1–U6, urban), the district of Sapucaia (T7–T10, transitional), and Patrocínio de Caratinga (P1–P20, rural).

To identify *Leishmania*, both sandflies and human samples were subjected to PCR. From a total of 13,043 specimens of sandflies collected, 1,054 pools were obtained, 936 in the collection carried out in 2020 and 118 in 2021. The sandfly collection carried out in 2020 in Patrocínio de Caratinga showed a total of 13 pools (∼1.23%) positive for *L. braziliensis* from *Ny. whitmani* (7), *Ny. intermedia* (4), and *Mg. migonei* (2) pools. Positive pools were detected at points P3 (5), P4 (1), P10 (2), P12 (1), P13 (2), P16 (1), and P20 (1). None of the 118 pools collected in 2021 in the city of Caratinga and in the urban area of the district of Sapucaia were positive. Not all areas with high density of sandflies had high density for infected sandflies (Figure 8F). Between 2020 and 2021, 27 human biopsies (19 men and 8 women) were obtained from the Caratinga Health Service. In all samples, *L. braziliensis* was identified as the species causing ATL. Cytochrome B gene was amplified in 40 of the 7,547 females from *Ny. whitmani* (15), *Ny. intermedia* (14), *Mg. migonei* (10), and *Ev. lenti* (1), all from Patrocínio de Caratinga. Blood from five vertebrate species was detected (Table 6). *Homo sapiens* was the most common blood meal source (40%), and *Ny. whitmani* had blood of all five vertebrate species.

## DISCUSSION

In this study, an additional spatial analysis (2019–2019) following a previously published historical series (2007–2018)^5^ is provided. We also collected sandflies in the municipality in urban and rural areas affected by ATL. Those sandflies and biopsies from human patients were screened for the presence of *Leishmania*. Also, blood meal sources were investigated to understand sandfly preference habits in the area.

Confirming our previous data,^5^ ATL cases were consistently reported in the last triennium (2019–2021), although the frequency of their occurrence did not vary much from 2016–2018 (13.82 versus 11.77). Males are the most vulnerable population (70%) and the demographic aspects of ATL transmission did not change in the last period, as reported elsewhere.^5^ In Minas Gerais, as in several Brazilian regions, ATL is a predominantly rural disease.^23^^–^^25^ However, an increase in the number of cases reported in the urban area can be observed in the last period. This is of importance because in this work we confirmed that ATL presence is also expanding toward other rural and urban areas.

Analyzing the entire period (2007–2021), there was a noticeable fluctuation in the number of cases of ATL with peaks in 2010–2012 and 2016–2018 periods.^5^ In 2019 and 2020, ATL cases were still high declining in 2021. The low number of cases observed this year may be the result of several factors derived from underreporting, a fact that is commonly also observed in other studies.^26^^–^^28^ Since the beginning of our study, Patrocínio de Caratinga and Sapucaia districts recorded the most rural cases since 2007.^5^ In 2019–2021, in addition these districts, Santa Luzia de Caratinga recorded a high number of rural cases. More important, a high frequency of urban cases was detected in the past 3 years (22.94%) compared with the historical series (19.75%) and with the last triennium. This increase may be due to several factors, including displacement of rural populations to urban areas and vice versa, migratory flows of the parasite reservoirs, and the adaptation of vector species to urban areas. Because there was no homogeneity in the incidence rates along time, the high maintenance of ATL cases in the last 3 years confirms urban and rural transmission of the disease. Although we cannot exclude that COVID-19 pandemics may have affected ATL underreporting in 2021, it is clear that the return of the ATL Reference Center led to an improvement of diagnosis.

Spatial analysis provides valuable information for decision-making and policy development in health and disease prevention.^29^ One of the limitations of our study is the size and shape of the municipality of Caratinga (Figure 1) and the distance of some districts from the urban area. This leads some patients to seek care in nearby cities, hindering notification. On the basis of our previous spatial analysis, the urban Caratinga and the rural Patrocínio de Caratinga and Sapucaia were the most affected by ATL.^5^ However, changes in incidence over time can be explained by variations in transmission patterns resulting from climatic and environmental conditions.^30^^,^^31^ It is also necessary to consider the bias of reported cases. In the period 2019–2021, the cases of ATL reported in the municipality of Caratinga showed a homogeneous distribution even compared with the previous triennium (2016–2018). However, the higher incidence rates as well as the expansion of the disease to other regions warrant prevention and control measures in such areas.

Consistent with this observation, the 2019–2021 ellipse comprised a larger area and reinforced expansion of ATL not only in the urban but also in the rural areas, with hotspots in the rural districts of Patrocínio de Caratinga/Sapucaia and in the urban area of Caratinga. In the last two trienniuns (2016–2018/2019–2021), the hotspots coverage increased in the aforementioned areas but also in other areas of the municipality. As previously noted, together with migration, agricultural practices, deforested areas, and disordered human occupation also can contribute to ATL transmission by favoring proximity to the vectors.^32^ The urban area of Caratinga had hotspots since 2007, reinforcing that ATL is already urbanized there for a long time. In the last triennium evaluated, not only the central area of the city but also the entire southern area had a greater coverage of cases. However, the RRs of the disease did not change for urban Caratinga and rural Patrocínio de Caratinga. However, a higher number of newly reported urban cases compared with rural cases in that district reinforce the need for ATL surveillance and control measures in these areas.

Entomological surveys in the municipality of Caratinga are scarce and date from the 1970 s.^6^^,^^7^ They were carried out in other areas, some of which no longer belong to the municipality and were subjected to drastic environmental changes. Here, we provided more detailed entomological data and molecular identification of infection and blood source. The species *Ny. whitmani*, *Ny. intermedia*, and *Mg. migonei* were most abundant, proven vectors of ATL. These species, widely distributed throughout South America, can tolerate and even overcome environmental changes, especially those caused by human action, adapting to survive new ecological niches.^33^^,^^34^ The diversity of sandfly species had higher abundance in Patrocínio de Caratinga, showing their adaptability to anthropic actions.^35^ In urban environments, the presence of vegetation serves as habitats for breeding and resting places, especially peridomestic sites including vegetation, roots, tree trunks, and organic matter.^36^^–^^38^

The entomological captures were in three ecological sites. Those variations were confirmed by the diversity parameters assessed and are related to the conservation levels of such ecotopes. Thus, they may affect availability of food and refuge for sandflies.^33^ The richness of sandfly species is higher in forested areas than in less forested areas.^39^^,^^40^ This is in agreement with the greater richness of sandflies observed in the rural area of Patrocínio de Caratinga in relation to urban Caratinga and Sapucaia district.

Several epidemiological studies have detected *Leishmania* infection in sandflies.^41^^,^^42^ Similar to other studies, we found low detection of *Leishmania* infection (∼1.23%). However, after molecular identification, the species *L. braziliensis* was detected only in the sandflies from rural Patrocínio de Caratinga. On the other hand, we could not find positive sandflies in urban areas of Caratinga and Sapucaia. However, this may be a result of a lower number of insects captured because the presence of *Leishmania* is low in the field. Although the collection points in Sapucaia (T7–T10) were considered urban, these areas have rural characteristics and therefore should be better classified as transition areas. The presence of chicken coops and vegetable crops close to the domestic environment represent a risk factor for the creation and maintenance of a high density of vectors. Another hypothesis highlights that the diversity (H) of insects in the transition area can be justified by the location of this area between the forest and the urban environment. This fact probably led to a higher number of insects collected in Sapucaia compared with the city of Caratinga.^43^^–^^45^

In Brazil, the parasitological examination remains the standard for the diagnosis of ATL. However, these approaches are laborious and require trained staff. In this sense, molecular techniques are useful to help diagnosis. Like sandflies, *L. braziliensis* was also detected in the biopsies of the patients from urban and rural areas, confirming that the parasite is circulating in these locations.^46^^–^^48^

Finally, analysis of blood ingested by different species of sandflies provides important information for the study of *Leishmania* infection and transmission. We identified five species of mammals in 40 samples of sandflies. Interestingly, the species *Homo sapiens* was the main source of food detected in the four specimens that were engorged. This is of importance because it shows that the vectors have human preference increasing the chances of ATL transmission.^49^^,^^50^

In conclusion, this work demonstrates the diversity of sandflies with proven vectors of ATL with human preference for blood meal. We also detected *L. braziliensis* as the cause of ATL in the patients and the only species found in sandflies captured in the rural area of Patrocínio de Caratinga. In the last two trienniums, cases of ATL have been increasingly reported in the urban area of Caratinga and expanded toward other rural regions. Altogether, these data reinforce the need of epidemiological surveillance in the entire municipality of Caratinga to prevent serious future ATL outbreaks.
